# 1-Benzyl-3,5-bis­(2-thienylmethyl­ene)-4-piperidone

**DOI:** 10.1107/S1600536809033923

**Published:** 2009-09-05

**Authors:** Ju-feng Sun, Hong-juan Li

**Affiliations:** a Binzhou Medical College, Yantai 264003, People’s Republic of China

## Abstract

In the title compound, C_22_H_19_NOS_2_, the thio­phene rings form angles of 69.74 (18) and 65.56 (16)° with the benzene ring. The piperidone ring adopts a half-chair conformation due to the presence of the conjugated ketone systems. Both thio­phene rings are disordered over two orientations [occupancies of 0.758 (2)/0.242 (2) and 0.588 (2)/0.412 (2)] at 180° from one another. In the crystal, weak inter­molecular C—H⋯O hydrogen bonds, C—H⋯π and aromatic π–π stacking inter­actions [shortest centroid–centroid separation = 3.865 (3) Å] help to stabilize the packing.

## Related literature

For general background to 3,5-bis(arylidene)-4-piperidone derivatives, see: Baluja *et al.* (1964 ▶). Benvenuto *et al.* (1993 ▶); Dimmock *et al.* (1983 ▶); Dimmock *et al.* (2003 ▶); El-Subbagh *et al.* (2000 ▶). For details of the synthesis, see: Pati *et al.* (2009 ▶).
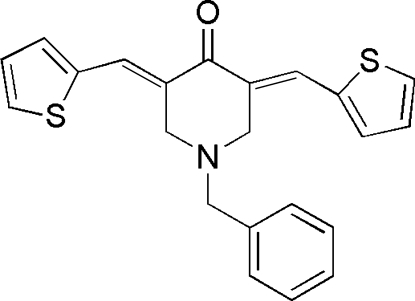


## Experimental

### 

#### Crystal data

C_22_H_19_NOS_2_
*M* *_r_* = 377.50
Triclinic, 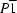

*a* = 5.7110 (11) Å
*b* = 9.4072 (19) Å
*c* = 17.338 (4) Å
α = 87.82 (3)°
β = 87.55 (3)°
γ = 81.99 (3)°
*V* = 921.1 (3) Å^3^
*Z* = 2
Mo *K*α radiation
μ = 0.30 mm^−1^
*T* = 113 K
0.19 × 0.16 × 0.10 mm

#### Data collection

Rigaku Saturn CCD area-detector diffractometer
Absorption correction: multi-scan (*CrystalClear*; Rigaku/MSC, 2005 ▶) *T*_min_ = 0.945, *T*_max_ = 0.971
9377 measured reflections
4321 independent reflections
3660 reflections with *I* > 2σ(*I*)
*R*_int_ = 0.031

#### Refinement

*R*[*F* ^2^ > 2σ(*F* ^2^)] = 0.039
*wR*(*F* ^2^) = 0.100
*S* = 1.05
4321 reflections
268 parameters
58 restraints
H-atom parameters constrained
Δρ_max_ = 0.33 e Å^−3^
Δρ_min_ = −0.23 e Å^−3^

### 

Data collection: *CrystalClear* (Rigaku/MSC, 2005 ▶); cell refinement: *CrystalClear*; data reduction: *CrystalClear*; program(s) used to solve structure: *SHELXS97* (Sheldrick, 2008 ▶); program(s) used to refine structure: *SHELXL97* (Sheldrick, 2008 ▶); molecular graphics: *SHELXTL* (Sheldrick, 2008 ▶); software used to prepare material for publication: *CrystalStructure* (Rigaku/MSC, 2005 ▶).

## Supplementary Material

Crystal structure: contains datablocks I, global. DOI: 10.1107/S1600536809033923/bg2287sup1.cif

Structure factors: contains datablocks I. DOI: 10.1107/S1600536809033923/bg2287Isup2.hkl

Additional supplementary materials:  crystallographic information; 3D view; checkCIF report

## Figures and Tables

**Table 1:** Hydrogen-bond geometry (Å, °)

*D*—H⋯*A*	*D*—H	H⋯*A*	*D*⋯*A*	*D*—H⋯*A*
C9—H9*A*⋯O1^i^	0.99	2.59	3.4946 (18)	151
C10—H10*B*⋯O1^i^	0.99	2.56	3.4747 (18)	153
C11—H11⋯O1^ii^	0.95	2.45	3.319 (2)	153
C13—H13⋯O1^ii^	0.95	2.56	3.338 (6)	140
C2—H2⋯*Cg*6^iii^	0.95	2.68	3.520 (5)	148

Symmetry codes: (i) 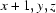; (ii) 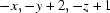; (iii) 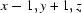.

**Fig. 1.**
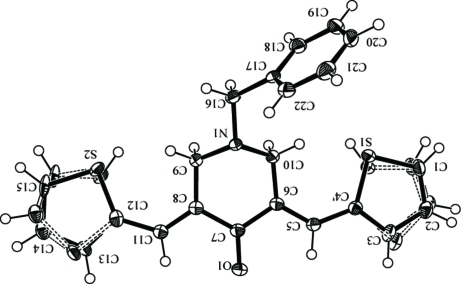
The molecular structure of (I), showing 50% probability displacement ellipsoids and the atomic numbering. Only the main part of each disordered thienyl ring is labelled.

**Fig. 2.**
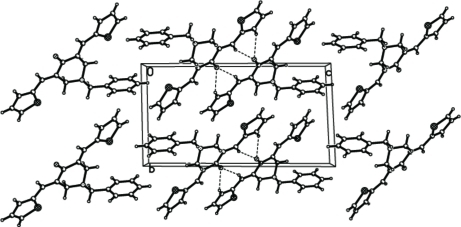
The packing diagram of (I). Dashed lines indicate C—H···O hydrogen bonds.

## supplementary crystallographic information

### Comment

At present, a series of 3,5-bis(arylidene)-4-piperidone derivatives have been synthesized and proved to display cytotoxic and anticancer properties (El-Subbagh, *et al.* 2000; Dimmock, *et al.* 2003). These compounds possess marked affinities for thiols but with little or no affinities for amino or hydroxyl groups found in nucleic acids (Baluja, *et al.* 1964; Dimmock, *et al.* 1983). Thus development of these compounds as candidate cytotoxics may lead to the obtention od drugs which lack the undesirable genotoxic properties present in various antineoplastic agents (Benvenuto *et al.* 1993). Here, we report the title compound (I), which is a combination of cyclic α, β-unsaturated ketone (chalcone) and β-amino ketone, which could be used as a basic unit to prepare antineoplastic compounds.

The molecular structure of the title compound (I) is shown in Fig. 1. The thiophene rings determine angles of 69.74 (18)° and 65.56 (16)° with the benzene ring. The piperidone ring adopts a half-chair conformation due to the presence of conjugated ketone systems,and both of the thiophene rings were found disordered over two orientations, respectively. In the crystal, weak intermolecular C—H···O hydrogen bonds and aromatic π-π stacking interactions [shortest centroid- centroid separation = 3.865 (3) Å] help stabilizing the packing.

### Experimental

The title compound was synthesized according to the literature (Pati *et al.* 2009). Dry hydrogen chloride was continuously bubbled into a solution of *N*-benzyl-4-piperidone (0.01 mol) and 2-thieneylaldehyde (0.02 mol) in acetic acid (25 ml) at room temperature. Then the mixture was stirred at room temperature for 8 h., when the precipitate obtained was collected and washed with acetone (20 ml) and added to an aqueous potassium carbonate solution (5%, *w*/*v*). The desired product was obtained after the solid was crystallized in a mixture of ethanol and chloroform (1:1, V/V), in a yield of 75.6%. Suitable crystals for X-ray analysis were obtained by slow evaporation of the solution of title compound in a mixture of chloroform and methanol.

### Refinement

All H atoms were positioned geometrically and refined in the riding model approximation with C—H = 0.95 and 0.99 Å. Both thiophene rings were found disordered with occupancies of 0.758 (2)/0.242 (2) and 0.588 (2)/0.412 (2), respectively. The disordered thiophene moieties were restricted to have C—C, Cδbond C and C—S distances of 1.46 (1)Å, 1.36 (1) Å and 1.7 (1) Å, respectively.

### Crystal data

**Table:**

C_22_H_19_NOS_2_	*Z* = 2
*M**_r_* = 377.50	*F*(000) = 396
Triclinic, *P*1	*D*_x_ = 1.361 Mg m^−^^3^
Hall symbol: -P 1	Mo *K*α radiation, λ = 0.71073 Å
*a* = 5.7110 (11) Å	Cell parameters from 2974 reflections
*b* = 9.4072 (19) Å	θ = 2.3–27.4°
*c* = 17.338 (4) Å	µ = 0.30 mm^−^^1^
α = 87.82 (3)°	*T* = 113 K
β = 87.55 (3)°	Block, colorless
γ = 81.99 (3)°	0.19 × 0.16 × 0.10 mm
*V* = 921.1 (3) Å^3^	

### Data collection

**Table:**

Rigaku Saturn CCD area-detector diffractometer	4321 independent reflections
Radiation source: rotating anode	3660 reflections with *I* > 2σ(*I*)
confocal	*R*_int_ = 0.031
Detector resolution: 7.31 pixels mm^-1^	θ_max_ = 27.9°, θ_min_ = 2.2°
ω and φ scans	*h* = −7→7
Absorption correction: multi-scan (*CrystalClear*; Rigaku/MSC, 2005)	*k* = −11→12
*T*_min_ = 0.945, *T*_max_ = 0.971	*l* = −22→22
9377 measured reflections	

### Refinement

**Table:**

Refinement on *F*^2^	Secondary atom site location: difference Fourier map
Least-squares matrix: full	Hydrogen site location: inferred from neighbouring sites
*R*[*F*^2^ > 2σ(*F*^2^)] = 0.039	H-atom parameters constrained
*wR*(*F*^2^) = 0.100	*w* = 1/[σ^2^(*F*_o_^2^) + (0.0449*P*)^2^ + 0.2715*P*] where *P* = (*F*_o_^2^ + 2*F*_c_^2^)/3
*S* = 1.05	(Δ/σ)_max_ = 0.002
4321 reflections	Δρ_max_ = 0.33 e Å^−^^3^
268 parameters	Δρ_min_ = −0.23 e Å^−^^3^
58 restraints	Extinction correction: *SHELXL97* (Sheldrick, 2008), Fc^*^=kFc[1+0.001xFc^2^λ^3^/sin(2θ)]^-1/4^
Primary atom site location: structure-invariant direct methods	Extinction coefficient: 0.062 (7)

### Special details

**Table:**

Geometry. All e.s.d.'s (except the e.s.d. in the dihedral angle between two l.s. planes) are estimated using the full covariance matrix. The cell e.s.d.'s are taken into account individually in the estimation of e.s.d.'s in distances, angles and torsion angles; correlations between e.s.d.'s in cell parameters are only used when they are defined by crystal symmetry. An approximate (isotropic) treatment of cell e.s.d.'s is used for estimating e.s.d.'s involving l.s. planes.
Refinement. Refinement of *F*^2^ against ALL reflections. The weighted *R*-factor *wR* and goodness of fit *S* are based on *F*^2^, conventional *R*-factors *R* are based on *F*, with *F* set to zero for negative *F*^2^. The threshold expression of *F*^2^ > σ(*F*^2^) is used only for calculating *R*-factors(gt) *etc*. and is not relevant to the choice of reflections for refinement. *R*-factors based on *F*^2^ are statistically about twice as large as those based on *F*, and *R*- factors based on ALL data will be even larger.

### Fractional atomic coordinates and isotropic or equivalent isotropic displacement parameters (Å^2^)

**Table:**

	*x*	*y*	*z*	*U*_iso_*/*U*_eq_	Occ. (<1)
O1	0.10269 (17)	1.04217 (11)	0.40042 (6)	0.0258 (2)	
N1	0.6906 (2)	0.83369 (11)	0.29936 (6)	0.0179 (2)	
S1	0.62468 (11)	1.24588 (7)	0.15845 (4)	0.02485 (18)	0.757 (2)
C1	0.5420 (7)	1.3962 (7)	0.1016 (3)	0.0266 (8)	0.757 (2)
H1	0.6425	1.4337	0.0632	0.032*	0.757 (2)
C2	0.3140 (7)	1.4554 (5)	0.1182 (4)	0.0254 (7)	0.757 (2)
H2	0.2357	1.5379	0.0920	0.031*	0.757 (2)
C3	0.2069 (10)	1.3777 (7)	0.1801 (4)	0.0271 (8)	0.757 (2)
H3	0.0490	1.4040	0.1993	0.033*	0.757 (2)
C4	0.3533 (11)	1.2632 (9)	0.2086 (6)	0.0188 (9)	0.757 (2)
S1'	0.1590 (8)	1.4029 (5)	0.1772 (3)	0.0271 (8)	0.243 (2)
C1'	0.351 (2)	1.4701 (19)	0.1149 (13)	0.0254 (7)	0.243 (2)
H1'	0.3151	1.5556	0.0842	0.031*	0.243 (2)
C2'	0.563 (3)	1.388 (2)	0.1139 (13)	0.0266 (8)	0.243 (2)
H2'	0.6929	1.4068	0.0808	0.032*	0.243 (2)
C3'	0.5729 (16)	1.2660 (11)	0.1688 (6)	0.02485 (18)	0.243 (2)
H3'	0.7127	1.2021	0.1794	0.030*	0.243 (2)
C4'	0.357 (3)	1.255 (3)	0.2029 (19)	0.0188 (9)	0.243 (2)
C5	0.2893 (2)	1.17132 (14)	0.27215 (8)	0.0193 (3)	
H5	0.1337	1.1968	0.2935	0.023*	
C6	0.4133 (2)	1.05527 (14)	0.30644 (7)	0.0178 (3)	
C7	0.3030 (2)	0.99300 (14)	0.37716 (8)	0.0187 (3)	
C8	0.4454 (2)	0.87146 (14)	0.41855 (7)	0.0182 (3)	
C9	0.6814 (2)	0.81022 (14)	0.38344 (7)	0.0190 (3)	
H9A	0.8074	0.8558	0.4061	0.023*	
H9B	0.7113	0.7059	0.3960	0.023*	
C10	0.6555 (2)	0.98827 (14)	0.27952 (8)	0.0198 (3)	
H10A	0.6743	1.0031	0.2229	0.024*	
H10B	0.7767	1.0351	0.3041	0.024*	
C11	0.3543 (2)	0.82590 (14)	0.48683 (7)	0.0197 (3)	
H11	0.2046	0.8765	0.5019	0.024*	
S2	0.7205 (3)	0.61663 (12)	0.53477 (8)	0.0240 (2)	0.5857 (19)
C12	0.445 (3)	0.716 (4)	0.5395 (19)	0.0203 (8)	0.5857 (19)
C13	0.3181 (9)	0.6735 (6)	0.6021 (3)	0.0232 (3)	0.5857 (19)
H13	0.1606	0.7155	0.6145	0.028*	0.5857 (19)
C14	0.4430 (6)	0.5605 (3)	0.64780 (19)	0.0247 (7)	0.5857 (19)
H14	0.3802	0.5200	0.6939	0.030*	0.5857 (19)
C15	0.6667 (8)	0.5177 (7)	0.6164 (4)	0.0290 (11)	0.5857 (19)
H15	0.7766	0.4427	0.6375	0.035*	0.5857 (19)
S2'	0.3013 (3)	0.64625 (16)	0.61623 (9)	0.0232 (3)	0.4143 (19)
C12'	0.469 (4)	0.710 (6)	0.540 (3)	0.0203 (8)	0.4143 (19)
C13'	0.6958 (16)	0.6399 (9)	0.5400 (5)	0.0240 (2)	0.4143 (19)
H13'	0.8182	0.6600	0.5041	0.029*	0.4143 (19)
C14'	0.7256 (14)	0.5307 (11)	0.6014 (6)	0.0290 (11)	0.4143 (19)
H14'	0.8709	0.4704	0.6095	0.035*	0.4143 (19)
C15'	0.5303 (10)	0.5224 (5)	0.6456 (3)	0.0247 (7)	0.4143 (19)
H15'	0.5202	0.4562	0.6880	0.030*	0.4143 (19)
C16	0.9204 (2)	0.76582 (15)	0.26799 (8)	0.0215 (3)	
H16A	0.9432	0.6628	0.2840	0.026*	
H16B	1.0479	0.8110	0.2900	0.026*	
C17	0.9401 (2)	0.77921 (14)	0.18092 (8)	0.0196 (3)	
C18	1.1212 (3)	0.84215 (15)	0.14380 (9)	0.0267 (3)	
H18	1.2351	0.8780	0.1734	0.032*	
C19	1.1376 (3)	0.85323 (17)	0.06335 (9)	0.0342 (4)	
H19	1.2626	0.8962	0.0383	0.041*	
C20	0.9723 (3)	0.80178 (18)	0.02021 (9)	0.0355 (4)	
H20	0.9831	0.8093	−0.0346	0.043*	
C21	0.7906 (3)	0.73904 (18)	0.05690 (9)	0.0329 (4)	
H21	0.6764	0.7038	0.0272	0.039*	
C22	0.7747 (3)	0.72757 (16)	0.13676 (8)	0.0251 (3)	
H22	0.6499	0.6841	0.1616	0.030*	

### Atomic displacement parameters (Å^2^)

**Table:**

	*U*^11^	*U*^22^	*U*^33^	*U*^12^	*U*^13^	*U*^23^
O1	0.0185 (5)	0.0275 (5)	0.0292 (5)	0.0011 (4)	0.0057 (4)	0.0033 (4)
N1	0.0179 (6)	0.0160 (5)	0.0182 (5)	0.0023 (4)	0.0023 (4)	0.0001 (4)
S1	0.0206 (4)	0.0249 (3)	0.0266 (3)	0.0014 (2)	0.0062 (2)	0.0067 (2)
C1	0.0339 (12)	0.0242 (11)	0.021 (2)	−0.0028 (9)	0.0019 (13)	0.0046 (10)
C2	0.0331 (16)	0.0182 (13)	0.0241 (10)	−0.0003 (12)	−0.0031 (13)	0.0014 (8)
C3	0.0295 (19)	0.0229 (17)	0.0278 (7)	0.0002 (12)	−0.0064 (11)	0.0057 (9)
C4	0.0208 (7)	0.0175 (12)	0.0176 (17)	−0.0004 (6)	−0.0005 (6)	−0.0038 (13)
S1'	0.0295 (19)	0.0229 (17)	0.0278 (7)	0.0002 (12)	−0.0064 (11)	0.0057 (9)
C1'	0.0331 (16)	0.0182 (13)	0.0241 (10)	−0.0003 (12)	−0.0031 (13)	0.0014 (8)
C2'	0.0339 (12)	0.0242 (11)	0.021 (2)	−0.0028 (9)	0.0019 (13)	0.0046 (10)
C3'	0.0206 (4)	0.0249 (3)	0.0266 (3)	0.0014 (2)	0.0062 (2)	0.0067 (2)
C4'	0.0208 (7)	0.0175 (12)	0.0176 (17)	−0.0004 (6)	−0.0005 (6)	−0.0038 (13)
C5	0.0174 (6)	0.0186 (6)	0.0218 (6)	−0.0022 (5)	0.0005 (5)	−0.0015 (5)
C6	0.0170 (6)	0.0171 (6)	0.0194 (6)	−0.0029 (5)	0.0005 (5)	−0.0030 (5)
C7	0.0178 (6)	0.0168 (6)	0.0214 (6)	−0.0023 (5)	0.0001 (5)	−0.0029 (5)
C8	0.0185 (7)	0.0174 (6)	0.0193 (6)	−0.0036 (5)	−0.0007 (5)	−0.0032 (5)
C9	0.0182 (6)	0.0192 (6)	0.0187 (6)	−0.0002 (5)	−0.0001 (5)	0.0006 (5)
C10	0.0192 (7)	0.0173 (6)	0.0216 (6)	0.0005 (5)	0.0025 (5)	0.0014 (5)
C11	0.0202 (7)	0.0189 (6)	0.0202 (6)	−0.0031 (5)	0.0001 (5)	−0.0030 (5)
S2	0.0215 (5)	0.0240 (5)	0.0242 (4)	0.0047 (3)	−0.0041 (3)	0.0019 (3)
C12	0.025 (3)	0.019 (2)	0.0171 (7)	−0.004 (3)	−0.002 (3)	−0.0014 (8)
C13	0.0233 (5)	0.0290 (7)	0.0169 (7)	−0.0053 (4)	0.0021 (4)	0.0048 (4)
C14	0.035 (2)	0.0210 (17)	0.0186 (8)	−0.0037 (13)	−0.0021 (14)	0.0007 (11)
C15	0.041 (3)	0.0247 (15)	0.021 (3)	0.000 (2)	−0.010 (2)	0.0045 (12)
S2'	0.0233 (5)	0.0290 (7)	0.0169 (7)	−0.0053 (4)	0.0021 (4)	0.0048 (4)
C12'	0.025 (3)	0.019 (2)	0.0171 (7)	−0.004 (3)	−0.002 (3)	−0.0014 (8)
C13'	0.0215 (5)	0.0240 (5)	0.0242 (4)	0.0047 (3)	−0.0041 (3)	0.0019 (3)
C14'	0.041 (3)	0.0247 (15)	0.021 (3)	0.000 (2)	−0.010 (2)	0.0045 (12)
C15'	0.035 (2)	0.0210 (17)	0.0186 (8)	−0.0037 (13)	−0.0021 (14)	0.0007 (11)
C16	0.0192 (7)	0.0220 (6)	0.0215 (7)	0.0042 (5)	−0.0006 (5)	−0.0021 (5)
C17	0.0187 (7)	0.0162 (6)	0.0218 (6)	0.0047 (5)	0.0023 (5)	−0.0008 (5)
C18	0.0243 (7)	0.0219 (7)	0.0327 (8)	−0.0012 (6)	0.0043 (6)	−0.0001 (6)
C19	0.0343 (9)	0.0281 (8)	0.0359 (9)	0.0034 (7)	0.0152 (7)	0.0090 (6)
C20	0.0445 (10)	0.0348 (8)	0.0204 (7)	0.0153 (7)	0.0056 (7)	0.0043 (6)
C21	0.0342 (9)	0.0361 (8)	0.0257 (7)	0.0073 (7)	−0.0061 (6)	−0.0059 (6)
C22	0.0225 (7)	0.0264 (7)	0.0252 (7)	0.0002 (6)	0.0018 (5)	−0.0024 (6)

### Geometric parameters (Å, °)

**Table:**

O1—C7	1.2301 (17)	C11—C12	1.413 (4)
N1—C9	1.4659 (16)	C11—C12'	1.497 (5)
N1—C16	1.4688 (17)	C11—H11	0.9500
N1—C10	1.4696 (16)	S2—C15	1.704 (4)
S1—C1	1.715 (3)	S2—C12	1.716 (6)
S1—C4	1.735 (3)	C12—C13	1.355 (6)
C1—C2	1.366 (4)	C13—C14	1.428 (5)
C1—H1	0.9500	C13—H13	0.9500
C2—C3	1.439 (6)	C14—C15	1.381 (5)
C2—H2	0.9500	C14—H14	0.9500
C3—C4	1.359 (5)	C15—H15	0.9500
C3—H3	0.9500	S2'—C15'	1.707 (5)
C4—C5	1.441 (3)	S2'—C12'	1.735 (7)
S1'—C1'	1.677 (9)	C12'—C13'	1.372 (8)
S1'—C4'	1.720 (9)	C13'—C14'	1.450 (7)
C1'—C2'	1.342 (9)	C13'—H13'	0.9500
C1'—H1'	0.9500	C14'—C15'	1.336 (6)
C2'—C3'	1.458 (8)	C14'—H14'	0.9500
C2'—H2'	0.9500	C15'—H15'	0.9500
C3'—C4'	1.361 (9)	C16—C17	1.5108 (18)
C3'—H3'	0.9500	C16—H16A	0.9900
C4'—C5	1.479 (9)	C16—H16B	0.9900
C5—C6	1.3484 (19)	C17—C18	1.385 (2)
C5—H5	0.9500	C17—C22	1.390 (2)
C6—C7	1.4917 (18)	C18—C19	1.395 (2)
C6—C10	1.5007 (18)	C18—H18	0.9500
C7—C8	1.4870 (18)	C19—C20	1.378 (3)
C8—C11	1.3515 (19)	C19—H19	0.9500
C8—C9	1.5031 (18)	C20—C21	1.384 (3)
C9—H9A	0.9900	C20—H20	0.9500
C9—H9B	0.9900	C21—C22	1.385 (2)
C10—H10A	0.9900	C21—H21	0.9500
C10—H10B	0.9900	C22—H22	0.9500
			
C9—N1—C16	109.11 (10)	C8—C11—C12	130.8 (5)
C9—N1—C10	110.22 (10)	C8—C11—C12'	126.7 (6)
C16—N1—C10	110.54 (11)	C8—C11—H11	114.6
C1—S1—C4	92.37 (16)	C12—C11—H11	114.6
C2—C1—S1	111.8 (2)	C12'—C11—H11	118.6
C2—C1—H1	124.1	C15—S2—C12	92.9 (2)
S1—C1—H1	124.1	C13—C12—C11	123.1 (5)
C1—C2—C3	111.8 (3)	C13—C12—S2	110.5 (3)
C1—C2—H2	124.1	C11—C12—S2	126.4 (4)
C3—C2—H2	124.1	C12—C13—C14	114.0 (4)
C4—C3—C2	113.5 (4)	C12—C13—H13	123.0
C4—C3—H3	123.3	C14—C13—H13	123.0
C2—C3—H3	123.3	C15—C14—C13	111.2 (3)
C3—C4—C5	123.9 (4)	C15—C14—H14	124.4
C3—C4—S1	110.5 (3)	C13—C14—H14	124.4
C5—C4—S1	125.6 (2)	C14—C15—S2	111.3 (3)
C1'—S1'—C4'	93.9 (4)	C14—C15—H15	124.3
C2'—C1'—S1'	111.4 (5)	S2—C15—H15	124.3
C2'—C1'—H1'	124.3	C15'—S2'—C12'	93.0 (3)
S1'—C1'—H1'	124.3	C13'—C12'—C11	130.6 (6)
C1'—C2'—C3'	112.9 (6)	C13'—C12'—S2'	110.3 (4)
C1'—C2'—H2'	123.5	C11—C12'—S2'	119.1 (5)
C3'—C2'—H2'	123.5	C12'—C13'—C14'	111.5 (5)
C4'—C3'—C2'	111.5 (6)	C12'—C13'—H13'	124.2
C4'—C3'—H3'	124.3	C14'—C13'—H13'	124.2
C2'—C3'—H3'	124.3	C15'—C14'—C13'	114.0 (5)
C3'—C4'—C5	130.6 (14)	C15'—C14'—H14'	123.0
C3'—C4'—S1'	109.9 (6)	C13'—C14'—H14'	123.0
C5—C4'—S1'	116.2 (8)	C14'—C15'—S2'	111.3 (4)
C6—C5—C4	130.75 (18)	C14'—C15'—H15'	124.4
C6—C5—C4'	128.6 (4)	S2'—C15'—H15'	124.4
C6—C5—H5	114.6	N1—C16—C17	112.48 (11)
C4—C5—H5	114.6	N1—C16—H16A	109.1
C4'—C5—H5	116.6	C17—C16—H16A	109.1
C5—C6—C7	117.13 (12)	N1—C16—H16B	109.1
C5—C6—C10	124.42 (12)	C17—C16—H16B	109.1
C7—C6—C10	118.43 (11)	H16A—C16—H16B	107.8
O1—C7—C8	121.69 (12)	C18—C17—C22	118.98 (13)
O1—C7—C6	120.77 (12)	C18—C17—C16	121.17 (13)
C8—C7—C6	117.54 (11)	C22—C17—C16	119.85 (13)
C11—C8—C7	117.02 (12)	C17—C18—C19	120.50 (15)
C11—C8—C9	124.41 (12)	C17—C18—H18	119.8
C7—C8—C9	118.55 (11)	C19—C18—H18	119.8
N1—C9—C8	111.58 (11)	C20—C19—C18	119.98 (15)
N1—C9—H9A	109.3	C20—C19—H19	120.0
C8—C9—H9A	109.3	C18—C19—H19	120.0
N1—C9—H9B	109.3	C19—C20—C21	119.85 (14)
C8—C9—H9B	109.3	C19—C20—H20	120.1
H9A—C9—H9B	108.0	C21—C20—H20	120.1
N1—C10—C6	110.42 (11)	C20—C21—C22	120.19 (16)
N1—C10—H10A	109.6	C20—C21—H21	119.9
C6—C10—H10A	109.6	C22—C21—H21	119.9
N1—C10—H10B	109.6	C21—C22—C17	120.50 (15)
C6—C10—H10B	109.6	C21—C22—H22	119.7
H10A—C10—H10B	108.1	C17—C22—H22	119.7
			
C4—S1—C1—C2	−1.8 (8)	C9—N1—C10—C6	−63.56 (14)
S1—C1—C2—C3	1.4 (9)	C16—N1—C10—C6	175.74 (11)
C1—C2—C3—C4	−0.1 (12)	C5—C6—C10—N1	−150.83 (13)
C2—C3—C4—C5	178.1 (10)	C7—C6—C10—N1	31.04 (16)
C2—C3—C4—S1	−1.2 (13)	C7—C8—C11—C12	−180 (3)
C1—S1—C4—C3	1.7 (10)	C9—C8—C11—C12	−1(3)
C1—S1—C4—C5	−177.6 (10)	C7—C8—C11—C12'	−177 (4)
C4'—S1'—C1'—C2'	−2(3)	C9—C8—C11—C12'	1(4)
S1'—C1'—C2'—C3'	−2(3)	C8—C11—C12—C13	−173 (2)
C1'—C2'—C3'—C4'	6(4)	C8—C11—C12—S2	7(6)
C2'—C3'—C4'—C5	−166 (3)	C15—S2—C12—C13	0(3)
C2'—C3'—C4'—S1'	−7(4)	C15—S2—C12—C11	−179 (4)
C1'—S1'—C4'—C3'	5(3)	C11—C12—C13—C14	−180 (3)
C1'—S1'—C4'—C5	167 (3)	S2—C12—C13—C14	0(4)
C3—C4—C5—C6	−180.0 (8)	C12—C13—C14—C15	−1(3)
S1—C4—C5—C6	−0.7 (15)	C13—C14—C15—S2	1.3 (7)
C3—C4—C5—C4'	117 (16)	C12—S2—C15—C14	−0.9 (19)
S1—C4—C5—C4'	−64 (14)	C8—C11—C12'—C13'	11 (9)
C3'—C4'—C5—C6	−20 (6)	C8—C11—C12'—S2'	−169 (2)
S1'—C4'—C5—C6	−177.4 (12)	C15'—S2'—C12'—C13'	−2(5)
C3'—C4'—C5—C4	100 (18)	C15'—S2'—C12'—C11	178 (5)
S1'—C4'—C5—C4	−57 (12)	C11—C12'—C13'—C14'	−178 (6)
C4—C5—C6—C7	173.6 (8)	S2'—C12'—C13'—C14'	2(5)
C4'—C5—C6—C7	179 (2)	C12'—C13'—C14'—C15'	−1(4)
C4—C5—C6—C10	−4.6 (8)	C13'—C14'—C15'—S2'	−0.4 (13)
C4'—C5—C6—C10	1(2)	C12'—S2'—C15'—C14'	1(3)
C5—C6—C7—O1	3.41 (19)	C9—N1—C16—C17	177.35 (11)
C10—C6—C7—O1	−178.32 (12)	C10—N1—C16—C17	−61.29 (15)
C5—C6—C7—C8	−175.80 (12)	N1—C16—C17—C18	123.42 (14)
C10—C6—C7—C8	2.48 (18)	N1—C16—C17—C22	−56.69 (16)
O1—C7—C8—C11	−6.0 (2)	C22—C17—C18—C19	−0.1 (2)
C6—C7—C8—C11	173.18 (12)	C16—C17—C18—C19	179.75 (12)
O1—C7—C8—C9	175.57 (12)	C17—C18—C19—C20	0.2 (2)
C6—C7—C8—C9	−5.24 (18)	C18—C19—C20—C21	−0.1 (2)
C16—N1—C9—C8	−177.33 (11)	C19—C20—C21—C22	−0.1 (2)
C10—N1—C9—C8	61.11 (15)	C20—C21—C22—C17	0.2 (2)
C11—C8—C9—N1	155.81 (13)	C18—C17—C22—C21	−0.1 (2)
C7—C8—C9—N1	−25.90 (16)	C16—C17—C22—C21	−179.98 (13)

### Hydrogen-bond geometry (Å, °)

**Table:**

*D*—H···*A*	*D*—H	H···*A*	*D*···*A*	*D*—H···*A*
C9—H9A···O1^i^	0.99	2.59	3.4946 (18)	151
C10—H10B···O1^i^	0.99	2.56	3.4747 (18)	153
C11—H11···O1^ii^	0.95	2.45	3.319 (2)	153
C13—H13···O1^ii^	0.95	2.56	3.338 (6)	140
C2—H2···Cg6^iii^	0.95	2.68	3.520 (5)	148

Symmetry codes: (i) *x*+1, *y*, *z*; (ii) −*x*, −*y*+2, −*z*+1; (iii) *x*−1, *y*+1, *z*.
